# Effectiveness of mass trapping interventions using autocidal gravid ovitraps (AGO) for the control of the dengue vector, *Aedes* (*Stegomyia*) *aegypti*, in Northern Mexico

**DOI:** 10.1186/s13071-024-06361-y

**Published:** 2024-08-17

**Authors:** Jesús Alejandro Aguilar-Durán, Gabriel L. Hamer, Filiberto Reyes-Villanueva, Nadia Angélica Fernández-Santos, Sergio Uriegas-Camargo, Luis Mario Rodríguez-Martínez, José Guillermo Estrada-Franco, Mario Alberto Rodríguez-Pérez

**Affiliations:** 1 Instituto Politécnico Nacional, Centro de Biotecnología Genómica, Laboratorio de Biomedicina Molecular, Reynosa, Tamaulipas México; 2 Department of Entomology, Texas A&M University, College Station, TX USA; 3 Secretaria de Salud de Tamaulipas, Ciudad Victoria, México

**Keywords:** *Aedes aegypti*, Autocidal gravid ovitraps, Mexico, Surveillance, Vector control

## Abstract

**Background:**

Mosquito-borne diseases, such as malaria, dengue, Zika and chikungunya, pose significant public health threats in tropical and subtropical regions worldwide. To mitigate the impact of these diseases on human health, effective vector surveillance and control strategies are necessary. Traditional vector control methods, which rely on chemical agents such as insecticides and larvicides, face challenges such as resistance and environmental concerns. Consequently, there has been a push to explore novel surveillance and control tools. Mass trapping interventions have emerged as a promising and environmentally friendly approach to reducing the burden of mosquito-borne diseases. This study assessed mass-trapping interventions using autocidal gravid ovitraps (AGOs) on *Aedes aegypti* populations in Reynosa, Tamaulipas, Mexico.

**Methods:**

Four neighborhoods were selected to evaluate the effects of three treatments: AGO mass-trapping, integrated vector control (IVC), which included source reduction and the application of chemical larvicide and adulticide, and AGO + IVC on *Ae. aegypti* populations. A control area with no interventions was also included.

The effectiveness of the interventions was evaluated by comparing *Ae. aegypti* abundance between the pre-treatment period (9 weeks) and the post-treatment period (11 weeks) for each treatment.

**Results:**

Only treatment using AGO mass trapping with an 84% coverage significantly reduced *Ae. aegypti* female populations by 47%, from 3.75 ± 0.32 to 1.96 ± 0.15 females/trap/week. As expected, the abundance of *Ae. aegypti* in the control area did not differ from the pre- and post-treatment period (range of 4.97 ± 0.59 to 5.78 ± 0.53); *Ae. aegypti* abundance in the IVC treatment was 3.47 ± 0.30 before and 4.13 ± 0.35 after, which was not significantly different. However, *Ae. aegypti* abundance in the AGO + IVC treatment increased from 1.43 ± 0.21 before to 2.11 ± 0.20 after interventions; this increase may be explained in part by the low AGO (56%) coverage.

**Conclusions:**

This is the first report to our knowledge on the effectiveness of mass-trapping interventions with AGOs in Mexico, establishing AGOs as a potential tool for controlling *Ae*. *aegypti* in Northeastern Mexico when deployed with sufficient coverage.

**Graphical Abstract:**

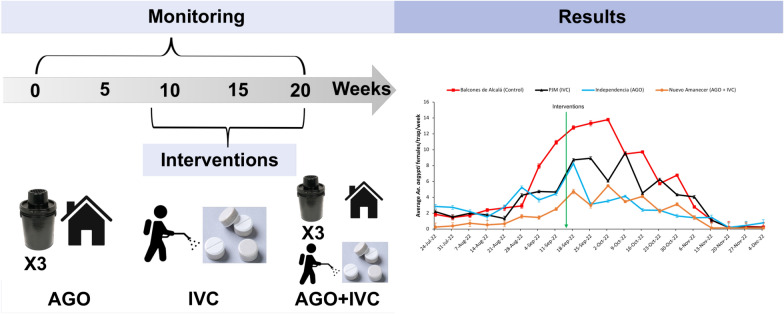

**Supplementary Information:**

The online version contains supplementary material available at 10.1186/s13071-024-06361-y.

## Background

*Aedes* (*Stegomyia*) *aegypti* is among the most studied mosquitoes because of its role in the transmission of pathogens such as yellow fever, dengue, Zika and chikungunya viruses [1]. The risk of *Ae*. *aegypti*-borne arbovirus transmission is particularly high in the tropical and sub-tropical urban areas worldwide. This is attributed to abundant larval mosquito habitats in urban landscapes, especially in communities with low socioeconomic conditions which include elevated number of abandoned properties, urban decline and improper waste management practices [2, 3]. In Mexico, *Ae*. *aegypti* is considered the primary vector of dengue [4], Zika and chikungunya viruses [5, 6]. The Asian tiger mosquito, *Aedes* (*Stegomyia*) *albopictus*, is considered a potential vector of these arboviruses [7]. These two species co-exist in much of the tropical and subtropical regions of the world, with *Ae. albopictus* extending further into temperate regions [8, 9].

In Mexico, dengue, Zika and chikungunya have coexisted since 2015 [10], with dengue being the most prevalent and widespread, affecting 29 out of 32 States [11]. All four dengue virus serotypes circulate in Mexico, and over the last decade, the country has ranked as the second highest in Latin America in terms of dengue cases, second only to Brazil [12]. In the state of Tamaulipas, which borders the USA, an estimated 91,665 probable cases of dengue and 2966 cases of dengue hemorrhagic fever were reported between 2012 and 2023 [13]. Furthermore, there were a total of 823 reported cases of Zika in Tamaulipas between 2015 and 2023 [14].

Control of vector mosquitoes typically involves residual spraying of homes and surrounding areas with adulticides, treatment of peri-domestic water containers using larvicides or larval habitat source reduction [15]. However, these strategies have been insufficient for the sustained control of *Aedes* mosquitoes and reduction of human *Aedes*-borne diseases. A contributing factor to the challenge is the rapid development of insecticide-resistance populations of *Ae. aegypti* in diverse parts of Mexico [16–18]. The widespread rise of insecticide resistance has led to the search for new alternatives to control vector dengue mosquitoes to reduce of the reliance on chemical control [19].

The autocidal gravid ovitrap (AGO), developed by the United States Centers for Disease Control and Prevention (CDC), is an environmentally friendly, sticky, lethal ovitrap designed for surveillance and control of female gravid *Aedes* mosquitoes [20]. The implementation of AGOs in mosquito control interventions has been carried out mainly in Puerto Rico, showing positive outcomes by effectively decreasing mosquito densities in mass-trapping interventions in both small [21, 22] and large areas [23]. Furthermore, studies conducted in other countries such as Colombia and the US have demonstrated variable effectiveness of these traps for *Ae. aegypti* surveillance and control [24–27].

Here, we evaluated the effectiveness of AGO mass trapping on *Ae. aegypti* populations in an urban area of Northern Mexico. Our results confirm the utility of AGOs as an alternative control tool for *Ae. aegypti* populations in endemic neighborhoods of the US-Mexico border region.

## Methods

### Study areas

The city of Reynosa (704,767 inhabitants; 216, 207 houses; INEGI 2020) is located at 26°03′03.0ʺN, 98°17′52.4ʺW, 30 m above sea level, in the state of Tamaulipas, Mexico. This is a sister city to McAllen, Texas, in Hidalgo County, USA. Reynosa has a hot dry climate with an average temperature of 22 ºC; during the “*canicula*” event in the dry season, which lasts about 40 days, daily temperatures can reach 40 to 42 ºC during the months of July–August. During the winter, in the months of December–February, the minimum temperature ranged from 0 to 5 ºC [28]. The study was conducted from July to December 2022 in four urban areas of about 100 households/area. The choice of this period was based on the bimodal local seasonality of *Ae. aegypti* (March–June and September–November) in the region, where the highest abundance occurs in the autumn [29], concurrent with when human disease from *Aedes*-borne viruses are observed [30].

**Fig. 1:**
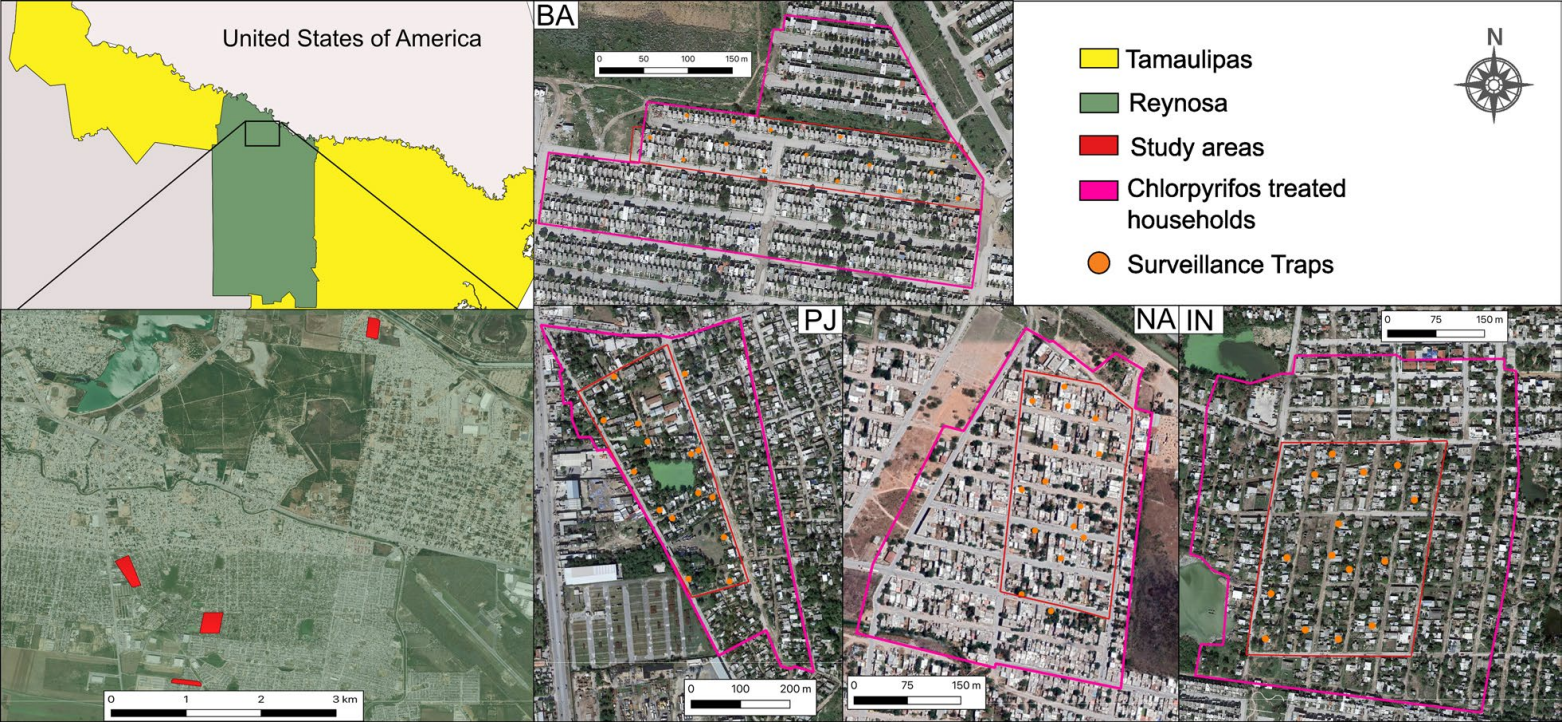
Study areas where SAGOs indicated by orange filled dots circles were deployed in Reynosa, Northern Mexico. The four areas are depicted individually and delineated by red lines: PJ: Pedro José Méndez, NA: Nuevo Amanecer, BA: Balcones de Alcalá, IN: Independencia; experimental households within each area were treated with Chlorpyrifos and cleaned; water-holding containers serving as breeding sites were removed. Map made with QGIS 3.16.6 (https://qgis.org/en/site/) and incorporated public domain map data from the Instituto Nacional de Estadística, Geografía e Informatica (National Institute of Statistics, Geography, and Computer Science [INEGI]; https://www.inegi.org.mx/app/mapas/), along with satellite images obtained from Google Maps (https://www.google.com/maps)

The four areas, Pedro José Mendez (26˚1′3.576ʺN, 98˚16′30.18ʺW), Balcones de Alcalá (26°00′08.1ʺN, 98°16′05.8" W), Independencia (26°00′41.3ʺN, 98°15′49.2ʺW) and Nuevo Amanecer (26°03′11.4ʺN, 98°14′33.3ʺW), were selected for this study (Fig. 1). Urban areas were chosen based on their history of recurrent dengue cases and their classification as low-income areas [29]. These areas are characterized by poor infrastructure, situated in semiurban areas with mostly one-story houses. Most streets are unpaved, with inadequate waste management practices and sewage systems. The streets frequently flood after rainfall, indicating poor storm water management with drains.

### Experimental design

**Fig. 2:**
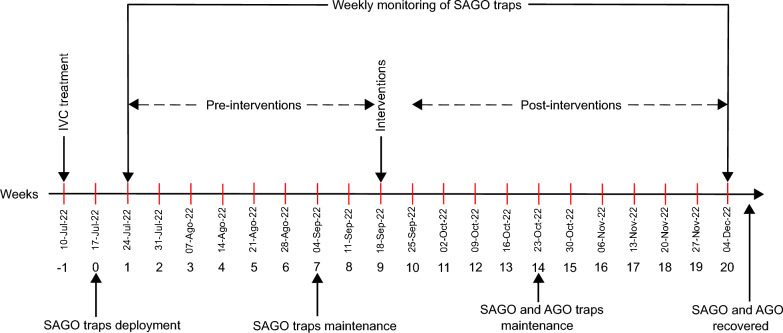
Study timeline. IVC treatment: source reduction, larviciding and application of ultra-low volume adulticide (Chlorpyrifos), SAGO: sentinel autocidal gravid ovitraps

A repeated-measures design was used to assess the impact of three treatments, the placement of three AGOs, an IVC approach which included source reduction, larvicide (Spinosad) and adulticide (Chlorpyrifos) application, and a combination of both treatments (AGO + IVM), and a control area, on the reduction of *Ae. aegypti* female populations, comparing the temporal changes in female densities in the four selected areas (neighborhoods). These four areas were randomly assigned to each treatment. The experimental design included a 9-week pre-treatment period from July to September 2022 and an 11-week post-treatment period from September to December 2022 (Fig. 2).

In each area, approximately 100 houses per area were selected to assess the treatment effect. Balcones de Alcalá (103 houses) was randomly designated as the control area, Independencia (116 houses) received the AGO trap treatment, Pedro José Méndez (105 houses) received the IVC treatment, and Nuevo Amanecer (109 houses) received the AGO + IVC treatment.

Before commencing the study, the inhabitants of each area were informed about the scope and objectives of the study. The initial approach to the inhabitants for placing the AGO traps was attempted during the morning or afternoon. If this attempt was unsuccessful, a second attempt was made during the weekend. In cases where initial contact was not successful or the resident declined to participate in the study, we continued to approach the next household until we obtained verbal consent.

In addition, a series of actions were carried out aiming to reduce the presence of *Ae. aegypti* and achieve a low and similar population in the four evaluated areas. One week prior to the start of sentinel autocidal gravid ovitrap (SAGO) deployment, an IVC treatment was performed on all four sites. This IVC treatment was performed in a 2–3 street buffer around the core areas (Fig. 1).

To monitor the abundance of *Ae. aegypti* females, 60 AGOs served as SAGOs with 15 deployed in each of the four study areas. The SAGOs were placed in the front yard of the randomly selected houses, ensuring a maximum distance of 100 m between each trap (Fig. 1). The monitoring of *Ae. aegypti* female populations during the pre-treatment period took place on a weekly basis from July 24 to September 18, 2022 (9 weeks).

At the end of the pre-intervention period, the treatments (AGO, IVC, AGO + IVC) were implemented (Fig. 2). The treatment with AGO traps consisted of placing three traps in the front and backyard of most of the selected houses in the area (56–84%), except for houses with a SAGO trap, where only two AGOs were deployed. The IVC treatment included source reduction and the application of chemical larvicide and adulticide, while in the control area, only the SAGOs were maintained.

The weekly post-treatment sampling period was from September 25 to December 4, 2022 (11 weeks). Preventive maintenance of AGOs was carried out every 2 months, involving removing external dirt and replacing grass infusions and sticky glue boards. All captured mosquitoes in SAGOs were visually identified to species [31], sexed and graded as fed and unfed based on the status of their abdomen.

### Statistical analysis

To examine whether there was a significant interaction of captured *Ae*. *aegypti* females between traps and weeks, we conducted an analysis of covariance (ANCOVA) using the MIXED procedure with a first-order autoregressive structure variance [32]. ANCOVA allowed us to control for potential covariates that might influence mosquito abundance, which is a crucial step in repeated measures studies, enabling a more robust assessment of the effects of interest and a more solid interpretation of the results obtained [33]. In this repeated measures study, the experimental unit was the SAGOs, with the number of females captured as the dependent variable and the location and trap number as random factors.

Additionally, to evaluate the effect of the treatments (AGO, IVC, AGO + IVC) on *Ae. aegypti* abundance, we contrasted the mean of *Ae. aegypti* females per SAGO during the pre-intervention period (9 weeks) compared to the post-intervention period (11 weeks). A multivariate negative binomial regression model was used, where the four treatments and time (pre and post) were considered predictors as class variables. The goodness of fit of the statistical model was evaluated using Pearson’s chi-square test for degrees of freedom (*χ*^2^/*df*), where values < 2 indicated a good fit of the model.

Differences between treatments and time were examined using the GLIMMIX procedure [34], designed to evaluate the effect of categorical, discrete or continuous variables with any probability distribution upon a count as a response variable. Additionally, comparisons between treatment and the control areas were calculated as a relative reduction in female *Ae. aegypti* by a modification of Henderson’s formula [35]:

$$\left(\text{\% Reduction}= \left(1-\left(\frac{{\text{Treatment}}_{t}}{{\text{Treatment}}_{Pre}}/\frac{{\text{Control}}_{t}}{{\text{Control}}_{Pre}}\right)\right)*100\%\right)$$

where subscripts denote the mean of females at time *t* and the pre-treatment mean and control area [36]. Also, a comparison of least square means was performed using Student’s *t* tests.

All statistical analyses were conducted using SAS OnDemand for Academics [37].

## Results

Ninety-three households of 105 from Pedro José Mendez (IVC area) were treated (88% coverage); 98 of 116 households from Independencia (AGO area) were treated (84% coverage; 294 AGOs), while in Nuevo Amanecer (AGO + IVC), only 62 of 109 households were treated (56% coverage; 186 AGOs) because many houses are abandoned; thus, the access to properties for deploying AGOs was not possible. Balcones de Alcala served as a control area with no treatment.

The results of the covariance analysis (Cov = − 0.0559; *χ*^2^ = 1.53, *df* = 1, *P* = 0.2167) indicate no significant interaction in the number of captured *Ae*. *aegypti* females between traps or weeks, regardless of the treatments. Therefore, the female abundance was independent between treatment and weeks.

The average number of *Ae. aegypti* females/trap/week in Balcones de Alcalá ranged from 4.97 ± 0.59 before interventions to 5.78 ± 0.53 after interventions. In Pedro José Mendez (IVC area), it was from 3.47 ± 0.30 to 4.13 ± 0.35, respectively. In Independencia (AGO area) from 3.75 ± 0.32 to 1.96 ± 0.15, respectively; and in Nuevo Amanecer (AGO + IVC area) from 1.43 ± 0.21 to 2.11 ± 0.20, respectively.

**Fig. 3:**
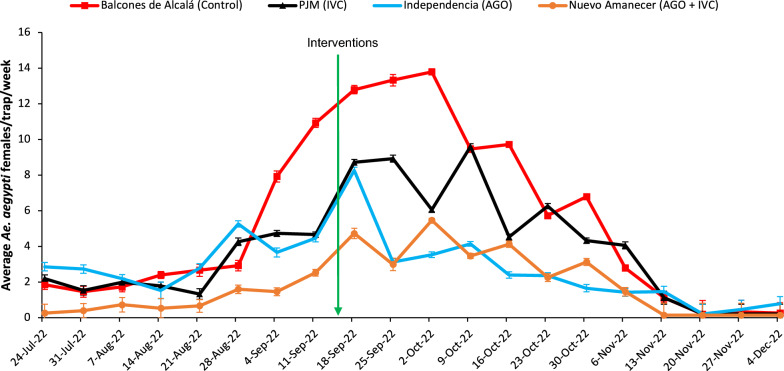
Weekly seasonal dynamics of *Aedes aegypti* females caught in SAGO traps. Balcones de Alcalá (control area), Pedro José Méndez (IVC area), Independencia (AGO area) and Nuevo Amanecer (AGO + IVC area). Values shown are least squares means

Only AGO treatment (Independencia) resulted in a significant decrease in *Ae. aegypti* female population in the pre- and post-intervention periods (GLIMMIX: *F*_(1, 298)_ = 29.10, *P* < 0.0001) indicating a reduction of 47% (Fig. 3). Contrarily, AGO + IVC treatment (Nuevo Amanecer) resulted in a higher number (2.11 ± 0.20 /trap/week) of females caught in SAGOs post-intervention (GLIMMIX: *F*_(1, 298)_ = 5.20, *P* = 0.0233) than that (1.43 ± 0.21 /trap/week) of pre-intervention. However, for IVC treatment (Pedro José Méndez), the female population was slightly reduced in the second week after interventions, but during the following weeks, that increased (GLIMMIX: *F*_(1, 298)_ = 1.70, *P* = 0.1935). As expected, the population did not vary (GLIMMIX: *F*_(1, 298)_ = 0.91, *P* = 0.3404) in the control area (Balcones de Alcalá).

Compared to the pre-treatment period, the overall reduction percentage in Pedro José Mendez (IVC area) was –2.3%, in Independencia (AGO area) it was 54.9%, and in Nuevo Amanecer (AGO + IVC area) it was –26.5% compared to the control area over the 11-week post-treatment period. Multiple comparisons of least square means revealed significant differences among all treatments (Additional file 1: Table S1).

## Discussion

Among the three treatments we evaluated, only the AGO intervention (deploying three AGOs/household with 84% coverage of area) reduced the female *Ae. aegypti* by 47% when comparing the mean number of females/trap/week. Additionally, it achieved a reduction of 54.9% when compared against control area using this repeated measures study design. Previous studies [38, 39] have assessed mass trapping interventions as an alternative strategy for controlling dengue vector mosquito populations. These studies have shown the efficacy of various types of traps, including AGOs [21–23, 40], standard lethal ovitraps [41, 42] and BG-GATs, to suppress *Ae. aegypti* populations [43]. However, some interventions, like those assessing three MosquiTRAPs (a sticky ovitrap) [44] or three BG-sentinel traps per household, failed to reduce adult *Ae. aegypti* populations [45].

Similar interventions with AGOs (placing three AGO traps per home) have proven effective at reducing *Ae. aegypti* female populations in Puerto Rico by 70% [21] and 79% [22] compared to the control areas. Furthermore, larger scale interventions in a medium-sized city from Puerto Rico demonstrated that a decrease in female population could be achieved when trap coverage exceeded 60% [23]. Our study reinforces the utility of AGOs for the control of *Ae. aegypti* populations.

The IVC intervention initially showed a slight reduction in the number of *Ae. aegypti* females after the second week, but it was not steady. Overall, it did not effectively reduce the female population. In contrast, the AGO intervention (Independencia) decreased the *Ae. aegypti* population, and this decline was steady for the rest of the study. The most recent reports on resistance to insecticides or larvicides in the Tamaulipas state have indicated susceptibility to those used in our study, namely Spinosad and Chlorpyrifos [46, 47]. Hence, insecticide resistance may not have influenced the results of our study. Surprisingly, we were expecting the intervention treatment in Nuevo Amanecer (AGO + IVC) to reduce the *Ae. aegypti* abundance. Contrarily, the female population was even higher post-AGO + IVC intervention than that of pre-intervention. This outcome can only be attributed to the lower AGO coverage (56%) in the selected area, primarily because of the many abandoned houses where it was impossible to deploy AGOs. As reported in Juarez et al. [27], this inadequate coverage may result in insufficient adult control by the traps, limiting the effect of these interventions. Furthermore, a recent study in Puerto Rico demonstrated the influence of vacant, abandoned or uninhabited buildings on the productivity of *Ae. aegypti*, where mosquito pupae were abundant in abandoned and inhabited houses. This highlights their significant contribution to increasing mosquito densities, resulting in ineffective control of vector mosquitoes [48]. 

The influence of non-residential areas on mosquito densities during field evaluations is a critical factor to consider. Diverse breeding sites within these areas provide suitable habitats for mosquito larvae, contributing to their proliferation [49–51]. This could play a significant role in the maintenance of *Ae*. *aegypti* populations after treatments or interventions. Mosquitoes from these non-residential areas could then migrate (i.e. ‘spillover’) to study areas, potentially confounding the outcomes of the treatments. 

In Puerto Rico, a similar intervention (source reduction, larviciding and placing 3 AGOs per house) [40] with a coverage of at least 80% of the area resulted in 92% and 84% reduction of the *Ae. aegypti* female population in the two intervened sites, respectively.

The level of coverage thus plays a crucial role in decreasing mosquito populations using mass trapping interventions as shown here. Evaluations conducted in Puerto Rico have shown that a coverage of ≥ 60% is necessary to effectively decrease *Ae. aegypti* populations in interventions using AGOs [21–23]. Similar effects have also been observed in interventions with BG-GATs, where areas with high coverage (≥ 80%) showed a significantly lower abundance of *Ae. albopictus* compared to areas with lower coverage (< 80) [43]. In contrast, interventions with low coverage (< 60%) using MosquiTRAPs [44] or BG-sentinel traps [45] did not result in a reduction of mosquito populations. In the present study, AGO coverage was only 56% in Nuevo Amanecer (AGO + IVC), which did not reduce the *Ae. aegypti* population; however, the AGO coverage in Independencia was 84% and thus significantly decreased the mean number of *Ae. aegypti* females.

The cost of mosquito traps, especially in mass trapping interventions, is an important factor to consider. According to Barrera et al. [21], the material cost for an AGO is USD$ 12.5. The SpringStar Biocare® Autocidal Gravid Ovitrap is sold commercially for USD$ 60 per two pack [52] but this price can be reduced for research and public health applications. This cost could be a limiting factor, especially in countries with a limited budget for mosquito control, where this may restrict the widespread adoption and long-term sustainability of mass trapping interventions. 

Moreover, understanding the fundamental ecology and biology of *Ae*. *aegypti*, from larvae to adults, is crucial for effective vector control. The knowledge encompassing breeding site preferences, feeding behavior and flight range across various environments allows us to predict mosquito populations and target interventions. Additionally, insights into adult mosquito behavior, such as host-seeking and resting habits, are vital for implementing strategies like insecticide application and trap placement, maximizing effectiveness and minimizing cost [53, 54]. Since AGOs are designed to capture and eliminate the adult stage, the larval life stage could persist. Therefore, it would be advisable to combine AGOs with a larval control method to achieve better results.

This is the first report on the effectiveness of mass trapping interventions with AGOs in Mexico. One limitation of the interventions was the inability to completely isolate the evaluated areas because this was conducted in an urban setting. Thus, the possibility of mosquito migration from non-intervention areas could not be ruled out, which could have influenced results. In addition, the interventions included only a single replicate of each treatment, and future studies should scale up the evaluation to include multiple replicates.

## Conclusions

This study highlights the potential of AGO mass trapping interventions as a valuable component of integrated vector management strategy for controlling *Ae. aegypti* populations. We found evidence of *Ae. aegypti* population suppression when AGO coverage was > 80%, confirming observations of prior work. However, a more comprehensive assessment of its effectiveness is necessary. Replication across diverse environmental conditions at multiple sites throughout different seasons is crucial. Furthermore, incorporating data on arboviral disease incidence and assessing the impact of mosquito populations from non-residential areas would provide more robust evidence regarding AGOs true potential, particularly as an alternative in areas where conventional adulticides are ineffective, such as those with insecticide resistance.

### Supplementary Information

Additional file 1: Table S1. Student’s t-test multiple comparisons of least squares means among the four treatments evaluated in the average number of female *Aedes* *aegypti* caught in SAGOs.

Additional file 2. *Aedes aegypti* database from SAGOs.

## Data Availability

The datasets employed and/or analyzed in the present study are available in the online supplementary material: Additional file 2: *Aedes aegypti* database from SAGOs.

## Abbreviations

AGO: Autocidal gravid traps
SAGO: Sentinel autocidal gravid traps
ICV: Integrated vector control
