# Supplementary material for: Effectiveness of mass trapping interventions using autocidal gravid ovitraps (AGO) for the control of the dengue vector, Aedes (Stegomyia) aegypti, in Northern Mexico
Source: Parasit Vectors. 2024 Aug 17;17:344. doi: 10.1186/s13071-024-06361-y (PMC11330617; doi:10.1186/s13071-024-06361-y)
Supplement: Supplementary file 1 — Additional file 1: Table S1. Student’s t-test multiple comparisons of least squares means among the four treatments evaluated in the average number of female Aedes aegypti caught in SAGOs. [file 13071_2024_6361_MOESM1_ESM.docx]

**Table S1.** Student's T test multiple comparisons of least squares means among the four treatments evaluated in the average number of female *Ae. aegypti* caught in SAGOs

| Treatment 1 | Treatment 2 | DF | | t Value | Pr > \|t\| |  |
| --- | --- | --- | --- | --- | --- | --- |
| AGO | AGO + IVC | | 1195 | 3.97 | <0.0001 | |
| AGO | IVC | | 1195 | -3.13 | 0.0018 | |
| AGO | Control | | 1195 | -6.55 | <0.0001 | |
| AGO+ IVC | IVC | | 1195 | -7.11 | <0.0001 | |
| AGO+ IVC | Control | | 1195 | -10.5 | <0.0001 | |
| IVC | Control | | 1195 | -3.46 | 0.0006 | |
